# The Wheat Head Blight Pathogen *Fusarium graminearum* Can Recruit Collaborating Bacteria from Soil

**DOI:** 10.3390/cells11193004

**Published:** 2022-09-26

**Authors:** Hina Ali, Mengtian Pei, Hongchen Li, Wenqin Fang, Hongkun Mao, Hamid Ali Khan, Tariq Nadeem, Guodong Lu, Stefan Olsson

**Affiliations:** 1 State Key Laboratory of Ecological Pest Control for Fujian and Taiwan Crops, College of Plant Protection, Fujian Agriculture and Forestry University, No. 15 Shangxiadian Road, Cangshan District, Fuzhou 350000, China; 2 Animal Genomics and Bioresource Research Unit, Faculty of Science, Kasetsart University, Bangkok 10900, Thailand; 3 Institute of Biological Sciences, Sarhad University of Science and Information Technology, Peshawar 25000, Pakistan; 4 Office of Research, Innovation and Commercialization, Bacha khan University Charsadda, Charsadda 24420, Pakistan; 5 National Centre of Excellence in Molecular Biology, University of Punjab, Lahore 53700, Pakistan; 6 Plant Immunity Center, Haixia Institute of Science and Technology, College of Life Science, Fujian Agriculture and Forestry University, No. 15 Shangxiadian Road, Cangshan District, Fuzhou 350000, China

**Keywords:** facultative endohyphal bacteria, fungal–bacterial interaction, symbiotic nitrogen fixation, new enrichment and isolation technique, gentamicin

## Abstract

In nature, fungal endophytes often have facultative endohyphal bacteria (FEB). Can a model plant pathogenic fungus have them, and does it affect their phenotype? We constructed a growth system/microcosm to allow an *F. graminearum* isolate to grow through natural soil and then re-isolated it on a gentamicin-containing medium, allowing endohyphal growth of bacteria while killing other bacteria. *F. graminearum* PH-1 labelled with a *His1mCherry* gene staining the fungal nuclei fluorescent red was used to confirm the re-isolation of the fungus. Most new re-isolates contained about 10 *16SrRNA* genes per fungal *mCherry* gene determined by qPCR. The *F. graminearum* + FEB holobiont isolates containing the bacteria were sub-cultured several times, and their bacterial contents were stable. Sequencing the bacterial *16SrRNA* gene from several Fg-FEB holobiont isolates revealed endophytic bacteria known to be capable of nitrogen fixation. We tested the pathogenicity of one common Fg-FEB holobiont association, *F. graminearum* + *Stenatrophomonas maltophilia*, and found increased pathogenicity. The *16SrRNA* gene load per fungal *His1mCherry* gene inside the wheat stayed the same as previously found in vitro. Finally, strong evidence was found for Fg-*S. maltophilia* symbiotic nitrogen fixation benefitting the fungus.

## 1. Introduction

Fungal–bacterial interactions are old and can be a simple competition for space and nutrients to more intimate interactions when bacteria grow on the surface of fungi, depending on the fungus for nutrients. Predatory bacteria can attack by destroying the cell walls [1] or parasitise fungi from the outside without destroying the fungal cell walls [2,3] or live inside the fungi as obligate or facultative symbionts [4,5,6,7]. Standard techniques to isolate fungi uses lab media containing a high concentration of nutrients and antibacterial antibiotics to suppress bacterial growth [8]. The side effect is that bacteria associated with the fungus in the natural environment are lost in culture.

Most fungi in nature could have obligate or facultative endohyphal bacteria (EHB), and such bacteria have been especially noted for endophytic fungi [9]. Endophytic/biotrophic growth is widespread in rhizosphere fungal phyla, like Basidiomycota, Glomeromycota, and Mucoromycota, and especially in the highly diverse Ascomycota [10]. Some notorious plant pathogens such as *F. graminearum* have an endophytic first stage before becoming necrotrophic. It has likely evolved as a beneficial endophyte of North American native grasses but is incompatible with wheat and other EuroAsian grasses [11]. EHB can influence the phenotypes of root-associated fungi and shape the outcomes of plant–fungal interactions [12]. EHB can be host-specific, vertically or horizontally transmitted, and can frequently maintain obligate relationships with fungi in the rhizosphere [13]. Associations between EHB and many endophytic species of Ascomycota are often with facultative endohyphal bacteria (FEB) since the bacteria can leave the hypha or re-enter the hyphae depending on conditions outside and inside the fungal cell [14]. Moreover, these FEB can be isolated from their fungal hosts by antifungal antibiotic treatment and are generally culturable on standard non-selective nutrient media [14]. However, functional relationships have been studied for only a few fungal–bacterial associations, limiting inferences regarding the scope and potential importance of EHB and FEB associations in ecological settings and human applications [4,15].

Bacteria can move by swimming, swarming, twitching, sliding, or gliding. Bacteria form a film around the fungal hyphae and disperse along fungal hyphae in natural environments, and the hyphae act as fungal highways for bacterial spread in ordinary aerated soil [16]. This hyphae-guided bacterial migration plays a vital role in the distribution of bacteria in soils. In vitro studies have also emphasized the ecological benefits of this dispersal [17]. However, evidence of active bacterial movement along hyphae in soils is still missing despite its potential ecological importance. Various approaches have been used to isolate soil bacteria moving along fungal hyphae selectively. One is a microcosm model based on a labelled Petri dish, pre-inoculated with *Lyophyllum* sp. Karsten strain to identify numerous bacterial species potentially moving along the fungal hyphae [18]. More recently, an inverted Petri dish method was used, in which *Pythium ultimum* (an Oomycete and not a fungus) mycelium was inoculated into the soil as a “fungal highway” to isolate contaminant-degrading bacteria [19]. Later, another approach was developed to isolate bacteria from the hyphae of filamentous fungi using fungal highway columns without pre-inoculation [7].

Our study aimed to find facultative endohyphal bacteria (FEB) present in soil with a previous history of soil presence of the well-characterized model *F. graminearum* wild-type strain PH-1 and to investigate the potential effect of FEB on growth and fungal plant pathogenicity. Several bacteria-containing isolates were found, and one potentially nitrogen-fixing FEB fungal–bacterial holobiont, Fg-*S. maltophilia*, was investigated in more detail.

## 2. Materials and Methods

### 2.1. Fungal Strains, Culture, and Growth Conditions

The well-studied *F. graminearum* wild-type strain PH-1 (NCBI: txid229533) was used. It was routinely grown on defined Fusarium medium (DFM) agar medium [20], with NO_3_ as the sole nitrogen source, consisting of glucose (12.5 g/L), NaNO_3_ (20 mM), MgSO_4_ 7H_2_O (2.1 mM), KH_2_PO_4_ (11.2 mM), KCl (7 mM), and trace minerals (Na_2_B_4_O_7_ 40 μg/L, CuSO_4_ 5H_2_O 400 μg/L, FeSO_4_ 7H_2_O 1200 μg/L, MnSO_4_ 1H_2_O 700 μg/L, NaMoO_2_ 2H_2_O 800 μg/L, ZnSO_4_ 7H_2_O 10 μg/L), and the pH set to 6.5 after autoclaving. For agar media, 2% agar was added. PH-1 and the FgHis1mCherry reporter strain were used as a non-bacterial background strain, and the Fg-FEB fungal strains containing FEB were grown, subcultured, and kept on DFM solid agar medium supplemented with gentamicin (0.512 mg/L). The fungal cultures were incubated at 24 °C with 12 h of light and dark cycles. For long-term storage, fungal cultures grown on small cut pieces of Whatman cellulose filter paper were dried and stored in sterilised envelopes with silica gel at 4 °C. To clarify, Table S1 contains descriptions and explanations for naming gene constructs and fungal strains containing bacteria with softened versions since this had to be introduced to avoid frequently repeated extended expressions.

### 2.2. Construction of Reporter Strain and Validation of (FgHis1mCherry) as Reporter Strain

A fungal reporter strain (FgHis1mCherry) was constructed by labelling the *F. graminearum* PH-1 with the vector *HIS1-mCHERRY-pCB1532*, previously constructed in our lab for the transformation of *Magnaporthe oryzae* [21]. The linker protein histone His1 in the relatively closely related *F. graminearum* is very similar. Positive fungal transformants were selected on DFM agar medium containing the selection marker G418 [21]. The FgHis1mCherry reporter strain was validated for the presence of the reporter gene. The gDNA isolated from the FgHis1mCherry reporter strain was amplified using PCR with the help of specific primers designed by Primer5 software (Table 1) and by employing the following PCR steps: initial denaturation for 5 min at 95 °C, denaturation for 15 s at 95 °C, annealing for 15 s at 58 °C, extension for 1 min and 30 s at 72 °C, and a final extension period for 10 min at 72 °C. The PCR product was run in a 1% agarose gel to check that the band for the FgmCherry reporter strain had the expected length. The eventual change in the morphology phenotype of the FgHis1mCherry reporter strain compared to PH-1 was evaluated on DFM agar medium plates and incubated at 28 °C for 5 days. The expression of FgmCherry protein in the reporter strain was validated through confocal microscopy for the presence of red fluorescent nuclei using a NikonA1 confocal microscope and 100× magnification (Plan Apo VC 100x Oil DIC, NA = 1.40), and it was also used to confirm that the hyphae were typically nucleated with 1 nucleus in most compartments except the tip compartments that contain more nuclei. Most importantly, the FgmCherry reporter strain was tested for conidiation, sexual reproduction, and with a pathogenicity assay, and compared with PH-1 to test that the random integration of the *HIS1-mCHERRY* construct had not disrupted any genes needed for wheat infection. 

### 2.3. Development of an Isolation Method to Catch FEB Using a Fungal Highway Column Technique

A fungal highway column with modifications to isolate FEB was developed based on an already described technique for selectively isolating bacteria moving along fungal hyphae [17], combined with using gentamicin to keep the FEB inside the hypha and kill external bacteria [22]. The fungal highway column was designed as a closed device (Ø: 15 mm, height: 48 mm) intended to select bacteria able to grow along or inside the fungal mycelium towards a target culture medium containing DFM + gentamicin, killing bacteria on the hyphal surface but leaving intracellular bacteria unharmed [22]. The test tube and lid were sterilised by immersion in 70% ethanol for 30 min and exposed to UV-C light for 30 min before assembling the system. Culture media and glass beads were sterilised by autoclaving (21 min at 121 °C). Each column was assembled aseptically inside a sterile laminar flow hood. At the bottom of the fungal highway column, as a first layer, sterile water agar was added to fill the rounded bottom of the column to make a flat support and was allowed to solidify. Then, a second thin layer of melted DFM medium was poured on top of the water agar and allowed to solidify and cool. A small mycelium plug of the FgHis1mCherry reporter strain was taken from a central part of the DFM agar plate with the help of a sterilised surgical blade and inoculated onto the center of the surface of the DFM agar medium. Next, a thin layer of sterilised 2 mm-diameter glass beads was added on top of the DFM agar disk with fungus to avoid direct contact with the soil added next. Then, a thin layer of sieved moist soil was added, followed by sterilised glass beads on the top of the soil in a several-centimeter-thick layer. A target medium, a DFM agar medium disk containing 0.512 mg/L gentamicin cut with a sterile cork borer from an agar plate, was added on top of the thick layer of washed and sterilised 2 mm-diameter glass beads. The now-prepared column was closed with the sterile cap for the tube. The column was incubated at 25 °C with a 12 h light and dark photoperiod. It was inspected regularly and allowed to incubate until the fungus had grown from the inoculum, through the soil and the thick glass-bead layer, and onto the target medium, and the fungal growth was visible on the target medium.

### 2.4. Isolation and Purification of Fg-FEB

The potential FEB moved along with the FgmCherry reporter hyphae and could be found in the target medium disk containing 0.512 mg/L gentamicin. The potential Fg-FEB was picked out of the column tube using a Pasteur pipette and employing a slight suction to catch the target agar disc. In that way, the DFM target agar discs, covered with fungus, were lifted out and inoculated onto a fresh agar medium plate with gentamicin (0.512 mg/L) poured into 10 cm-diameter Petri dishes by placing a disc against the agar in the center of the Petri dish and releasing the suction. The plates were incubated until the fungus had grown over most of the agar medium. For purification and to get facultative endohyphal bacteria, small plugs from the edges of potential Fg-FEB colonies were taken and subcultured on DFM agar plates with gentamicin (0.512 mg/L). These plates were incubated at 25 °C for 3–5 days with a 12 h light and dark photoperiod. After incubation, when the plates were covered with fungal growth, the potential Fg-FEB cultures were tested for the presence of bacteria.

### 2.5. Molecular Analysis of the Presence of FEB in the Potential Fg-FEB Cultures Using 16SrRNA Gene Sequencing

The primers for PCR of the mCherry gene were designed with the help of Primer5 software (Table 1). The conditions for PCR were: initial denaturation for 5 min at 95 °C, denaturation for 30 s at 95 °C, annealing for 30 s at 58 °C, extension for 1 min and 30 s at 72 °C, and final extension for 7 min at 72 °C. 

For *16SrRNA* gene sequencing, PCR universal primers for the bacterial *16SrRNA* gene were used for gene amplification. The PCR product was run in a 1% agarose gel to check the visible bands for the presence of FEB. After gel electrophoresis, the positive DNA samples were purified from the gel using a QIAGEN’s gel extraction kit and sent to QIAGEN, Fuzhou, Fujian, China, for *16SrRNA* gene sequencing. 

### 2.6. Comparison of Fg-FEB Colony Phenotypes with FgHis1mCherry

The colony morphology phenotype of the Fg-FEB cultures was compared with the background strain FgHis1mCherry cultures after growth on DFM agar medium with gentamicin (0.512 mg/L) at 25 °C for 4–5 days.

### 2.7. qPCR of FgFEBrRNA/His1mCHERRY to Estimate Bacterial Load in the Fungus

After confirming that the previously used primers performed well quantitatively for qPCR over a large range of diluted bacterial DNA together with fungal DNA (data not shown), the same primers were used for qPCR. Thus, to estimate the FEB load in the Fg-FEB strains, a qPCR procedure was used: initial denaturation at 95 °C for 2 min, followed by 40 cycles, denaturation at 95 °C for 15 s, annealing at 55 °C for 15 s, and extension at 72 C for 15 s. The amount of *His1mCHERRY* reporter gene was used as the “reference gene”, and the ratio of *16SrRNA* gene copies per *His1mCHERRY* reporter gene was an expression of the FEB load in the fungus. For qPCR SYBR premix, the Ex Taq II system (TaKaRa Perfect Real Time) was used. DNA isolated from respective Fg-FEB cultures was used as templates. 

### 2.8. Pathogenicity Assay of Fg-S. Maltophilia and PH-1 Used in Labs and Estimation of Fungal Bacterial Load during Wheat Coleoptile Infection

Wheat coleoptiles were infected with 10 μL conidia suspension (containing 4 × 10^4^ conidia) of Fg-*S. maltophilia* or PH-1 and incubated at 25 °C, with 12 h light and dark photoperiods, to determine the severity of coleoptile infection of the fungal isolates. The lesion size on infected wheat seedlings’ coleoptiles was measured at 7 dpi by photographing them with a ruler as a reference [23]. qPCR was used to estimate the bacterial load as *16SrRNA* gene copies per *mCherry* gene copies during wheat coleoptile infection when conidia or mycelia were used as inoculum. The bacterial load in the Fg-*S. maltophilia* grown in culture on DFM + gentamicin was used as a comparison. The qPCR was performed as above, and DNA isolated from the infected wheat coleoptiles was used as the template.

### 2.9. Testing Nitrogen-Fixing Capability of Fg-S. Maltophilia vs. PH-1 and Testing the Nitrogen-Fixing Ability by Comparing Protein Content of Cultures

A synthetic agar medium DFM—with double glucose content as the only added carbon source and no added nitrogen, supplemented with gentamicin at 0.512 mg/L—was used to keep the *S. maltophilia* from growing on the medium alone. The plates were incubated at 25 for 15–20 days with a 12 h light and dark photoperiod. The Bradford protein assay was used to determine the protein amount of Fg-*S. maltophilia* and PH-1 cultures on the medium without added a nitrogen source. Protein was extracted from a standardised area of the mycelium using a cork plug, and total proteins were extracted. The amount of Coomassie 1 Brilliant Blue G-250 dye colouring the extracted proteins (Bradford 1976) was then used to measure total protein content. A dilution series of bovine serum albumin (BSA) 2 mg/mL solution was used as a standard reference. The procedure followed the Quick Start Bradford Protein Assay Kit 1 (Bio-Rad, Beijing, China) protocol.

### 2.10. Statistics and Statistical Considerations

In this study, we often used ratios between two parameters measured for the same sample since that is a more stable comparison of these measured parameters and does not depend on random variations in sample biomass due to sampling errors. Since ratios of normally distributed measurements of parameters in the same samples are log-normally distributed, we chose to calculate the confidence intervals for the log-normal distribution but to present the bar plots with these ratios having a log2 *y*-axis labelled with the actual ratios. That way, the error bars are shown correctly and symmetrically around the averages, irrespective of the ratio values. As a final service to the reader, we used 95% confidence interval error bars combined with t-tests to control instead of ANOVA. This means that the readers can choose to compare whatever they find of interest, and we are also sure of not overinterpreting and assigning too low a probability for the null hypothesis of differences. Only averages with non-overlapping 95% error bars were considered significant, where *p* for the null hypothesis of no difference was < 0.05.

## 3. Results 

### 3.1. Validation of the FgHis1mCherry Reporter Strain 

**Figure 1:**
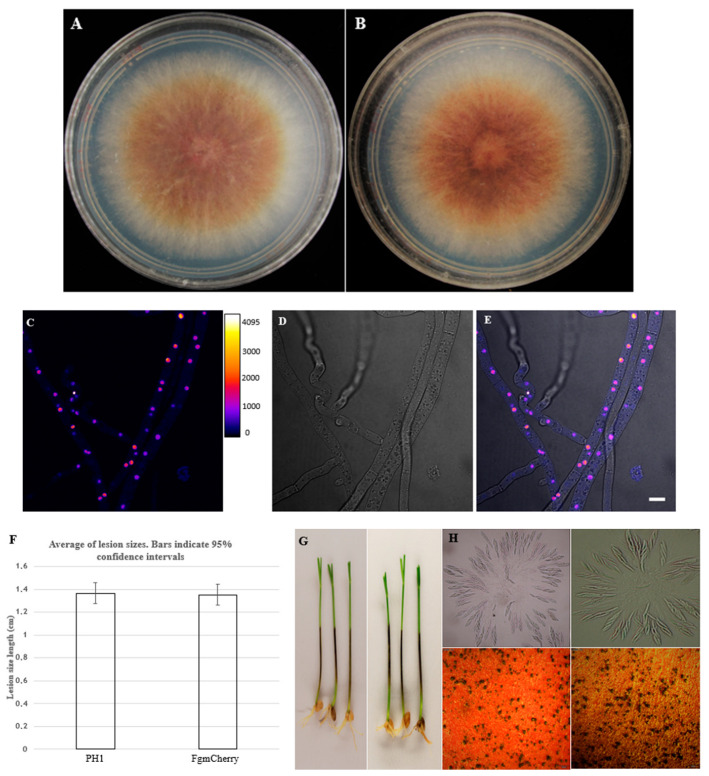
Validation of reporter strain FgHis1mCherry. (**A**) Wild-type PH-1 grown on DFM medium supplemented with gentamicin 0.512 mg/L to kill extracellular bacteria. (**B**) Compared with reporter strain FgHis1mCherry (FgmCherry). (**C**–**E**) Validation of reporter strain (FgHis1mCherry) shows the presence of the gene product of *HIS1mCHERRY* under a confocal microscope. The fluorescent red *Fusarium graminearum* is shown here using a Fire look-up table to show a large range of intensities of the confocal microscope (4096 levels) as temperature colors in the range of black, blue, red, orange, and white, which are better adapted to the human eye, revealing that His1mCherry mainly accumulates in the nuclei. (**F**) Comparison of lesion size (length) on wheat coleoptiles between PH-1 and FgmCherry. The error bar shows a 95% confidence level indicating that the bars with the overlapping error bars are not likely to be significantly different from the null hypothesis (*p* < 0.05) that they are the same, as also shown by the more conventional t-test (*t*-test *p* (same) = 0.85). (**G**) Wheat coleoptile lesions of PH-1 (left panel) and FgHis1mCherry (right panel). (**H**) Ascospore formation is normal for both PH-1 (top left panel) and for FgHis1mCherry (top right panel), as well as perithecia formation for PH-1 (bottom left panel) and FgHis1mCherry (bottom right panel). (The DIC image overlay has a 10 μm size bar.).

The FgHis1mCherry was validated using PCR, colony morphology, conidiation, sexual reproduction, and pathogenicity assay and compared with PH-1. The colony morphology, sexual reproduction, and pathogenicity assay of the FgHis1mCherry strain were similar to PH-1 (Figure 1A,B,F–H). Further, the validation of FgHis1mCherry was carried out by confocal microscopy. Fluorescent red fungal nuclei (displayed in the figure using a pseudo-colour gradient to highlight the accumulation in the nuclei) showed the presence of His1mCherry (Figure 1C–E).

### 3.2. Isolation and Purification of Fg-FEB Holobionts

**Figure 2:**
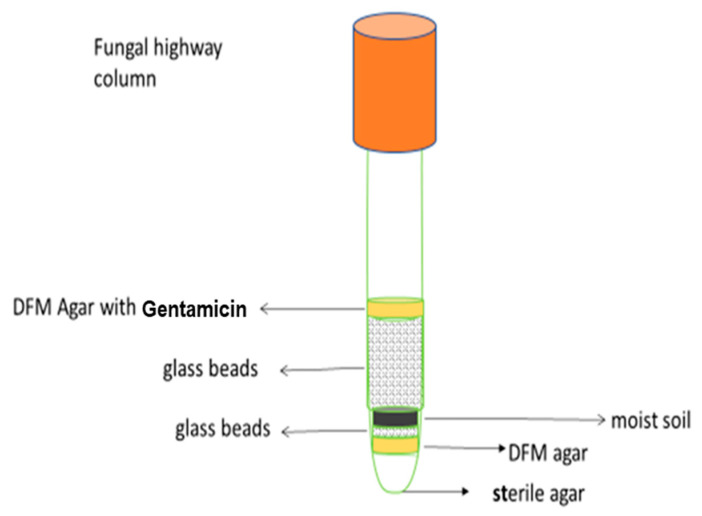
The fungal highway column technique to selectively isolate fungal-associated potential facultative endohyphal bacteria.

A modification of the fungal highway column [17] was constructed to obtain FEB coming along with fungal hyphae towards a target DFM medium supplemented with gentamicin (0.512 mg/L) (Figure 2) to isolate *F. graminearum* containing FEB [22]. After isolation on the gentamicin-containing DFM plates, the reporter strain still harboured FEB when the culture was fully grown on the DFM + gentamicin agar medium. The potential Fg-FEB were further subcultured for purification. The original FgHis1mCherry reporter strain could be reliably prescreened before PCR final confirmation (below) by using any system with green-light illumination and recording red fluorescence, since the white colony edge becomes fluorescent red in green light. We used our eyes and the indoor fluorescent light since the fungal colony edges become slightly pink to the eye in white light.

### 3.3. Molecular Detection of FEB Inside the FgHis1mCherry Reporter Strain

FEB inside the FgHis1mCherry strains were detected using PCR of the *16SrRNA* gene using the 16S universal primers for bacteria (Table 1). After detecting FEB inside the FgHis1mCherry strain, the positive isolates were sent for 16S DNA sequencing. After sequencing, the sequences were BLAST searched in the NCBI 16S sequence database. We found identities with several types of facultative endohyphal bacteria inside the FgHis1mCherry reporter strain. Some commonly occurring species were *Stenotrophomonas pavanii* (Fg-*S. pavanii*), *Stenotrophomonas maltophilia* (Fg-*S. maltophilia*, or Fg-Sm for short), and *Phytobacter ursingii* (Fg-*P. ursingii*). These are known as bacterial endophytes and, interestingly, capable of nitrogen fixation [24,25,26]. That is not an exhaustive list of all bacterial species that can be facultative endohyphal with *F. graminearum,* and we continued working with Fg-*S. maltophilia* since that was the most commonly identified species. 

### 3.4. Effect of FEB on FgHis1mCherry Colony Morphology on Standard Media

**Figure 3:**
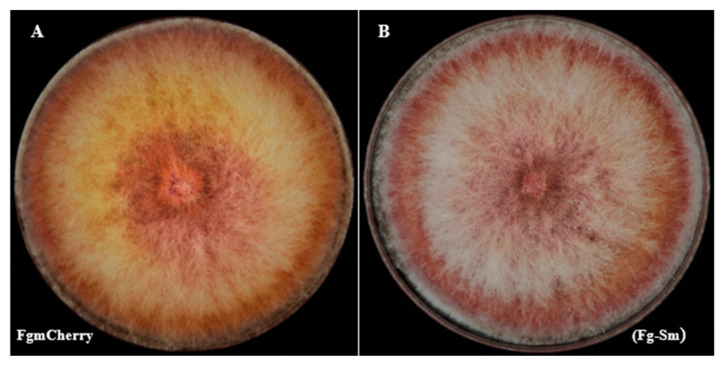
Morphology of FgHis1mCherry strain (FgmCherry) (**A**) compared with the Fg-*S. maltophilia* (Fg-Sm) isolate (**B**). Both were grown on DFM medium supplemented with gentamicin 0.512 mg/L.

Fg-*S. maltophilia* morphology was compared with the background FgHis1mCherry strain to check the FEB’s effect on the phenotype. The isolates were tested to evaluate the growth compared to the FgHis1mCherry fungal-only background. The result indicates that Fg-*S. maltophilia* has a similar colony phenotype as FgHis1mCherry, and the FEB has no marked effect on the overall colony phenotype on the medium tested (Figure 3). 

### 3.5. Stability of Fg-S. maltophilia and Estimation of Bacterial Load of the Fungus

After three months of subculturing (three times) on a DFM medium supplemented with gentamicin, the bacterial load was estimated as the number of *16SrRNA* genes per *mCherry* genes using qPCR. The subculturing was carried out in three separate lines of transfers between plates to estimate the eventual difference in the bacterial load in the fungus over time. The load stayed relatively constant, and we found it to be 10^1.18 (95% conf interval 0.567)^ =15 (11–41 95% confidence interval). When growing the Fg-*S. maltophilia* on DFM medium without gentamicin, bacterial colonies could be seen among the hyphae, while this was not the case for the sparse growth on distilled water agar without added nutrients.

### 3.6. Pathogenicity of Fg-S. maltophilia v/s PH-1

**Figure 4:**
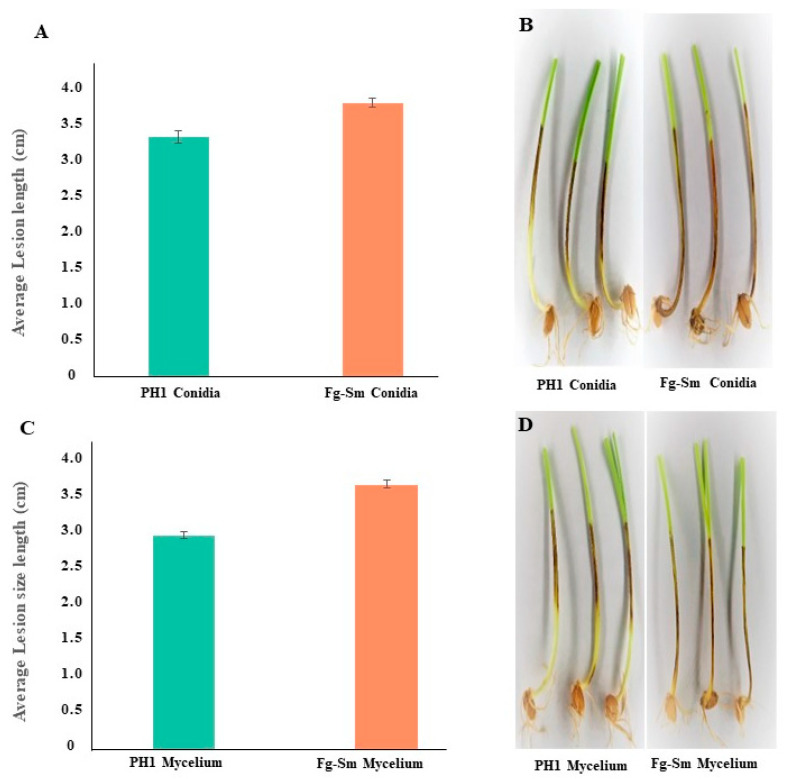
Pathogenicity assay of wheat coleoptiles infected with conidia and mycelium of the Fg-*S. maltophilia* (Fg-Sm) isolate and PH-1. (**A**) Lesion length measurements of coleoptiles infected with conidia (*t*-test same average *p* = 0.0010). (**B**) Coleoptiles with lesions infected with a conidial suspension of PH1 and Fg-Sm isolate. (**C**) Lesion length measurements of coleoptiles infected with mycelium (*t*-test same average *p* = 0.00011). (**D**) Coleoptiles with lesions infected with mycelium of PH1 and Fg-Sm isolate. Error bars show 95% confidence intervals. Non-overlapping confidence interval bars are significantly different (*p*_same_ < 0.05).

A pathogenicity assay using Fg-FEB was conducted and compared with PH-1. Wheat coleoptiles were infected with conidial suspension or mycelium discs of Fg-*S. maltophilia* and the wild-type PH-1. After incubation at 25 °C, the lesion size on the coleoptiles of infected wheat seedlings was measured after 7 days by photographing the seedlings with a ruler as a reference. Data were collected from three experiments’ lesion sizes of Fg-FEB and PH-1 [23]. Significant differences in lesion lengths were consistently observed (Figure 4A–D). The brown lesions’ lengths were used as an index for coleoptile infection severity and, consequently, the pathogenicity of the respective isolates. The lesion sizes were significantly longer for the Fg-*S. maltophilia* isolate than for the PH-1 (Figure 4A–D), indicating an FEB-increased pathogenicity.

### 3.7. Estimation of FEB Load in Fg-S. altophilia in Culture Conidia/Mycelium in Infected Plants

**Figure 5:**
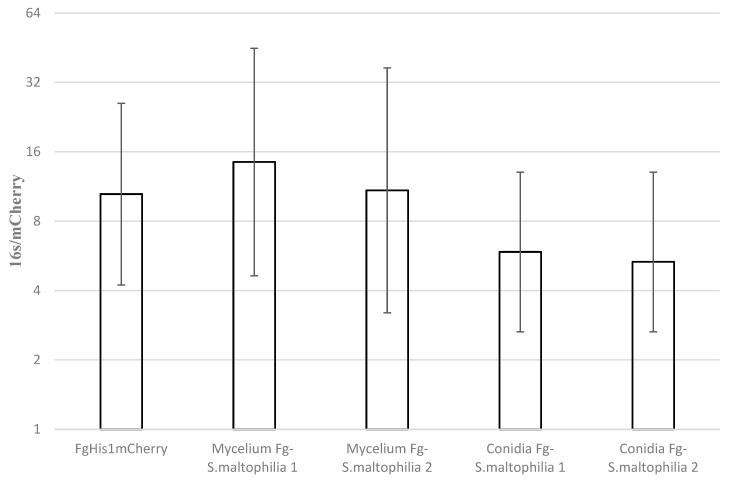
Estimation of *S. maltophilia* load in Fg-*S. maltophilia* during coleoptile infection. In vitro on gentamicin (FgHis1mCherry) containing DFM and inside wheat coleoptiles infected with conidia or mycelium of Fg-*S. maltophilia* isolates and bacterial load estimated using qPCR. Each inoculation method was repeated two times independently with three replicates each time. Error bars show 95% confidence intervals. Non-overlapping confidence interval bars are significantly different (*p* _same_ < 0.05). Pairwise *t*-test comparisons gave the following *p* values for the probability of different bacterial loads in the fungus in the plant infection compared to the in vitro-grown culture (FgHis1mCherry); mycelium inoculation Fg-*S. maltophilia* experiment 1 = 0.78, mycelium inoculation Fg-*S. maltophilia* experiment 2 = 0.98, conidia inoculation Fg-*S. maltophilia* experiment 1 = 0.61, conidia inoculation Fg-*S. maltophilia* experiment 2 = 0.57. Since the data are ratios, they are log-normally distributed, and all statistics were calculated on the log data values.

DNA was extracted from the wheat coleoptiles infected with conidia or mycelium of Fg-*S. maltophilia*. qPCR was performed to calculate the *16SrRNA* gene copies per fungal *His1mCherry*. Approximately 10 *16SrRNA* gene copies per fungal *His1mCherry* genes were found in the culture and coleoptiles when the plants were infected using mycelium as inoculum and around 6 when conidia were used as inoculum. The main reason to use this qPCR comparison was to avoid detecting negligible amounts of bacterial genomes compared to fungal genomes. These differences were insignificant, although there were significantly more *16SrRNA* gene copies than mCherry copies in the biomasses, thus the 95% error bars do not cross the 1/1 *X*-axis in Figure 5.

### 3.8. Nitrogen Fixation Capability of the Fg-S. maltophilia Holobiont

**Figure 6:**
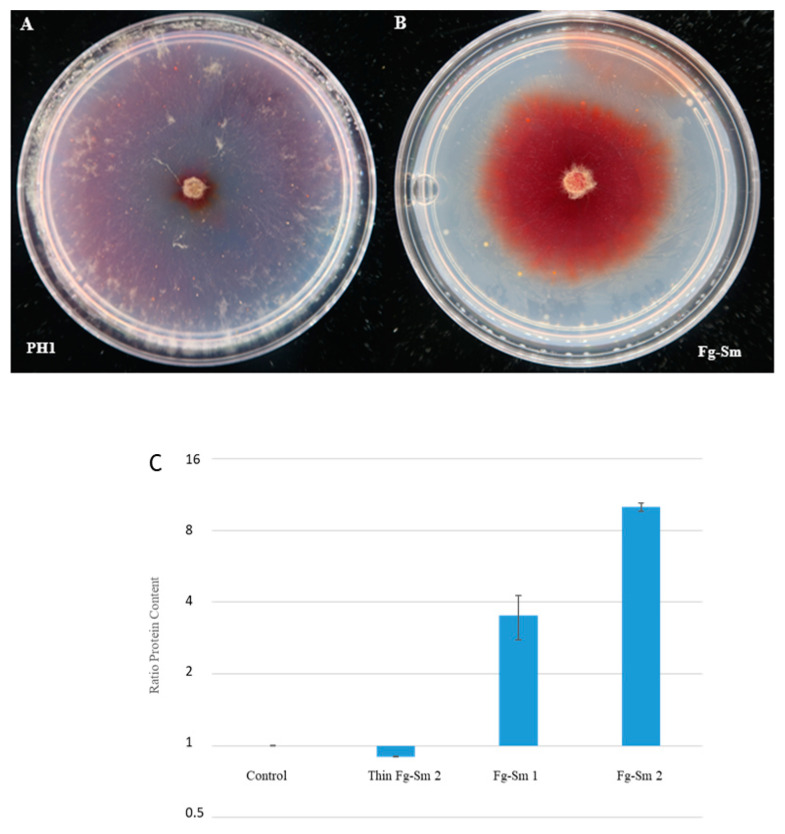
Nitrogen fixation capability of Fg-*S. maltophilia* vs. PH-1. Both cultures were grown on a nitrogen-free synthetic medium supplemented with gentamicin (0.512 mg/L) with only glucose as a carbon source and no nitrogen source. Plates were extracted for total protein determination when the control cultures had grown to the plate edge. (**A**) At that time, the thin growth of the Fg-*S. maltophilia* cultures had also reached the plate edge, as had the thin growth outer part of the Fg-*S. maltophilia* cultures. (**B**) The image is from a few days later of plates that had not been used for protein extraction to measure the protein content and confirmed that the dense red area expands over the thin area at the edge using the surplus carbon source that cannot be fully utilised without available nitrogen. (**C**) The ratio of protein content for Fg-*S. maltophilia* (or PH-1 control)/PH-1 control in different areas. Two independent experiments were used to confirm the high protein content of the Fg-*S. maltophilia* deep red colony areas (Fg-*S. maltophilia* 1 and 2). Error bars are 95% confidence intervals. Bars with non-overlapping error bars are significantly different (*p* _same_ < 0.05). Pairwise *t*-test comparisons gave the following *p* values for the relative protein content per area compared to the control (PH1): the thin external expanding growth of Fg-*S. maltophilia* = 0.74, Fg-*S. maltophilia* experiment 1 = 0.028, Fg-*S. maltophilia* experiment 2 = 0.0034. Since the data are ratios, they are log-normally distributed, and all statistics were calculated on the log data values.

We found several facultative endohyphal bacteria, for example, *S. maltophilia*, *S. pavanii*, and *P. ursingii*. All three are Gammaproteobacteria known as endophytes and, interestingly, are capable of nitrogen fixation [24,25,26]. The possible nitrogen fixation ability of the Fg-*S. maltophilia* holobiont was especially intriguing since that plant-growth-promoting rhizobacterium (PGPR) species has recently been proven to increase peanut nitrogen fixation [26] and to have a plant-growth-promoting effect on wheat under saline conditions [27]. The potential nitrogen-fixing capability of Fg-*S. maltophilia* was then tested by assessing its growth on a defined fungal agar medium without a nitrogen source added. The medium used was modified DFM with double the glucose concentration as a carbon source and no nitrogen source added. Gentamicin was, as previously, added at a 0.512 mg/L concentration to keep the FEB inside the Fg-*S. maltophilia.* Dark red, dense mycelium of Fg-*S. maltophilia* shows a high biomass, indicative of nitrogen fixation with a better yield on the medium with a single carbon source of glucose and a high formation of the secondary metabolite aurofusarin [28,29,30]. In the dark red area of the Fg-*S. maltophilia*, the measured total extractable protein per colony area was 3.5–10 times higher than in a comparable area of PH-1, indicative of the nitrogen fixation (Figure 6), while the protein content in the area outside the central dark red area (Figure 6B) was slightly lower than for PH-1 (Figure 6A). The slower radial growth of the dark red area also indicates a higher biomass yield per glucose added. Very thin and fast radial growth similar to PH-1 is seen for Fg-*S. maltophilia* outside the dark, red, and dense center area (Figure 6A,B), most probably indicating growth of the fungus with a low yield without N fixation, similar to PH-1. The red slower-growing center later expands to cover the plate. Slower radial growth with dense mycelium generally indicates a better yield on the carbon sources available in the agar [31,32,33].

### 3.9. Microscopy of the Fg-S. maltophilia Holobiont

**Figure 7:**
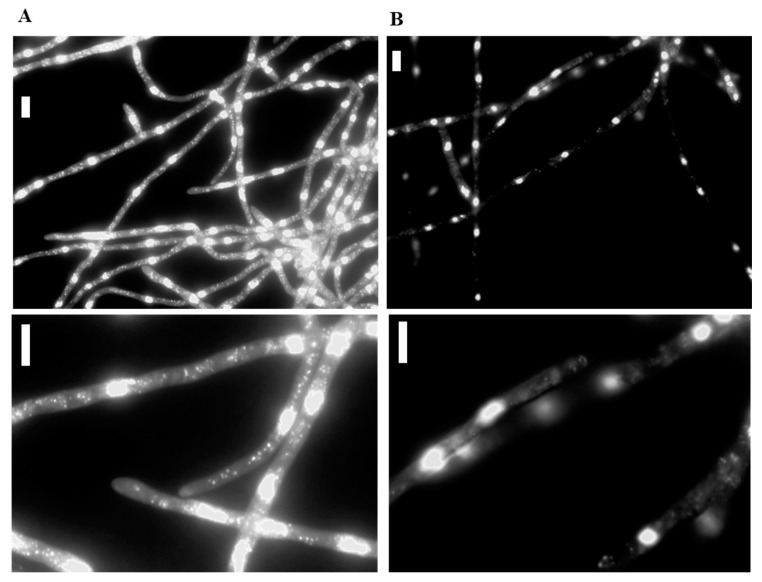
DAPI staining (blue, but shown in BW to give higher contrast for eyes) of hyphae with too long exposure to show nuclei well that contain a lot of DNA and become “overexposed” but can show bacteria and weakly also mitochondria that contain DNA. (**A**) With potential facultative endohyphal bacteria, (**B**) without potential facultative endohyphal bacteria. The lower images show higher magnification with hyphal tips where low-fluorescent active mitochondria should be positioned. These can be seen (**B** lower image). The bacteria containing hyphae (**A** lower image) are much brighter, thus it is difficult to see the mitochondria since the nuclei are very brightly stained and illuminate the cytoplasm that scatters light. That is why the cytoplasm is seen as a “darkfield” microscope illumination. Ordinary fluorescent microscopy using DAPI filters and 40× objective was used to have more hyphal biomass in one image and a deeper focal depth, since that facilitates detection of the bacteria in more of the cytoplasm. No bacteria were seen attaching to the edges of the hypha as is normally seen for bacteria associating with hyphal cell walls. Size bars = 20 μm.

To microscopically verify that the F. graminearum contains bacteria, we stained the hyphae grown in liquid DFM + gentamicin by mounting the hyphae in 1 mg/mL DAPI solution and visualised them using a standard fluorescent microscope to be able to see more hyphae in the same image and at a 1–2 μm hyphal depth. Initially, we had problems since the fungus did not take up DAPI unless we killed the hyphae by heating the microscope slide in a flame. To avoid heat destruction, we included an isotonic concentration of NaCN solution (0.15 M) in the mounting solution to inhibit ATP-dependent efflux pumps and avoid membrane destruction efficiently, and the staining was carried out in less than a minute. An abundance of tiny bright dots can be seen, indicating bacteria in the Fg-S. maltophilia hyphae (Figure 7A), while the parent strain that has not been in contact with soil has no such dots (Figure 7B). No bacteria were seen at the edges of the hyphae, as is usually the case with bacteria associating with hyphal cell walls externally [34] or even parasitising fungi without penetrating the cell walls [2].

## 4. Discussion

The soil bacteria we have found associated with *F. graminearum* as FEB belong to Gammaproteobacteria, a bacterial class commonly found as facultative endohyphal bacteria of the fungal class Sordariomycetes that *F. graminearum* and many fungal endophytes and plant pathogens belong to [9]. The FEB load inside the fungus was stable over time when using gentamycin to kill the bacteria escaping to grow on the medium [22]. We calculated the bacterial load to avoid considering contaminating, commensal, negligibly occurring bacteria as potentially endohyphal. In future experiments, when we have sequenced the strains and know precisely how many *16SrRNA* gene copies there are in the bacterium, this could—combined with a Southern blot of the fungus for control of the number of mCherry incorporations—possibly be translated to bacteria/fungal nuclei. Alternatively, the FEB can be labelled with a gene expressing GFP or using a unique DNA sequence amplified by specific primers. The copy number of *16SrRNA* varies between species [35] and with the growth of the bacteria, with higher copy numbers for faster growth rates in their natural environments [36]. The bacteria we found as FEB are reported to have around four copies per genome when grown in culture [35]. That should mean around 2.5 bacteria or more per fungal nuclei since the copy number of the mCherry gene in the fungal genome is at least one. This is also in rough agreement with what can be seen by microscopy (Figure 7). Since *F. graminearum* can be found in the soil plot at our university used for infection studies, it is probably a re-association of bacteria with the PH-1 strain we saw in our bacterial-free background fungal isolate, FgHis1mCherry, derived from PH-1.

Several of the bacteria we have found are known for their nitrogen-fixing ability. That applies to *S. maltophilia*, a known plant-growth-promoting bacterium [27] that can improve the nitrogen status of peanut plants [26]. We could show that the holobiont Fg-*S. maltophilia* can grow well and form dense mycelium with a high protein content on a defined agar medium without added nitrogen sources (Figure 6). Nitrogen is a limiting factor for plant colonization by plant pathogenic fungi, including *F. graminearum* [37]; thus, it is not surprising that the holobiont Fg-*S. maltophilia* showed slightly higher pathogenicity, although significantly higher than the background *F. graminearum* strain, FgHis1mCherry (Figure 4).

Interestingly, the load of Fg-*S. maltophilia* inside the fungal conidia and in the seedlings infected by mycelia were similar to what we found in culture (Figure 5). Thus, the FEB is, in this case, likely to be vertically transmitted, as has been shown for several endohyphal bacteria of Mucoromycota, and Glomeromycota, some of which are obligate endohyphal [6]. Consequently, we could expect to find many vertically transmitted FEB inside most types of fungi in nature if we looked for them and used isolation techniques allowing for a growing condition of the isolates that stop the bacteria from escaping and competing with the fungus as contaminants. Regular lab media [38] are usually much richer than the natural environments fungi and bacteria are adapted to live and grow in [33], including the so-called “rich” rhizosphere of plants [39]. When growing the Fg-*S. maltophilia* on distilled water agar only on nutrients available in the standard agar (not purified agar or agarose), no colonies could be seen on the agar, indicating that FEB intrahyphal growth could be occurring in nutrient-poor natural environments. These FEB were isolated and grown alone without fungi, and we sequenced two of the most common FEB isolates showing nitrogen-fixing ability. Since the genes responsible for nitrogen fixing in these genera are not well-described or easy to find by bioinformatics, we aim in the future to carry out TN5 mutagenesis and design a system for screening for critical genes needed for symbiotic N fixation together with *F. graminearum*, as well as to functionally test these when we know if we can transform these bacteria and re-introduce them to the fungus.

The natural state of *F. graminearum* soil and plant growth is thus probably an Fg-FEB holobiont. This holobiont can have a changed phenotype and ecological niche compared to *F. graminearum* without a bacterial partner. Most fungi might, in nature, contain endohyphal bacteria affecting the fungal physiology and ecology and the niche available for the fungal–bacterial holobiont. If so, we need to change our general practice for isolating, growing, and studying many fungi in the lab to better understand the physiology of fungi in their evolutionary natural state.

## Figures and Tables

**Table 1:** List of primers for mCherry and *16SrRNA* qPCR and *16SrRNA* sequencing.

Primers		Sequence
**mCherry**	**PCB1532 F**	**GCAACCCAAGGGACCATCT**
	**PCB1532 R**	**TTGACCTCAGCGTCGTAGTG**
***16SrRNA***	**27 F**	**AGAGTTTGATCCTGGCTCAG**
	**1492 R**	**TACGGTACCTTGTTACGACTT**

## Data Availability

The data presented in this study are available on request from the corresponding author. The data are not presently publicly available due to ongoing studies using partly the same data.

## Supplementary Materials

The following supporting information can be downloaded at: https://www.mdpi.com/article/10.3390/cells11193004/s1, Table S1: The naming of constructs and strains, including names for fungal-bacterial holobionts.
