# Supplementary material for: The Wheat Head Blight Pathogen Fusarium graminearum Can Recruit Collaborating Bacteria from Soil
Source: Cells. 2022 Sep 26;11(19):3004. doi: 10.3390/cells11193004 (PMC9563980; doi:10.3390/cells11193004)
Supplement: Supplementary file 1 [file cells-11-03004-s001.zip › cells-1915658-supplementary.pdf]

cells MDPI

Table S1. The naming of constructs and strains, including names for fungal-bacterial holobionts. Principles for naming genes and proteins are as normal for molecular biology. Genes in italic and proteins non-italic with 2 first letters indicating the fungal species. Fg= *Fusarium graminearum.* Mo= *Magnaporthe oryzae*. **The naming of holobiont components**: First two letters are the fungal species (in this case, Fg), then if probable Facultative Endohyphal bacteria are present after a hyphen FEB abbreviation. If the bacterial species have been identified, after the hyphen, the full bacterial name is in italic the first time and with only the initial letter for the genus as usual for species names.

Description	Short Form (in Figures)	Explanation
Fg containing FEBs	Fg-FEB	Short for the fungal species and FEBs of any bacteria. FEB since the endohyphal bacteria were acquired from the environment
Fg containing the *Histone 1* gene fused with the *mCherry* gene so that the strain can be recognized as containing the *mCherry* gene	*FgHIS1mCHERRY*	When we are referring to the gene construct or the gene expression. Gene in italic. The gene is, however the orthologous gene *MoHIS1mCHERRY* pointed out in methods.
Fg strain containing the histone 1 gene fused with the mCherry gene when discussing the mCherry protein	FgHis1mCherry (FgmCherry)	When referring to the reporter protein carrying strain. The protein is however a MoHis1mCherry.
Plasmids	*pCB1532*	Referring to the plasmid. DNA so gene in italic.
Fg containing:<br>Fg-*Stenotrophomonas pavanii*<br>Fg-*Stenotrophomonas maltophilia*<br>Fg-*Phytobacter urisingii*	Fg-*S. pavanii* (Fg-Sp)<br>Fg-*S. maltophilia* (Fg-Sm)<br>Fg-*P. ursingii* or (Fg-Pu)	If the Fg isolate is known to contain a specific bacterial species.
16SrRNA PCR primers<br>MomCHERRY PCR primers	*16SrRNA*<br>*mCherry*	PCR primer sequences
